# Study protocol: a systematic review of pediatric shared decision making

**DOI:** 10.1186/2046-4053-2-48

**Published:** 2013-07-01

**Authors:** Kirk D Wyatt, Gabriela Prutsky Lopez, Juan Pablo Domecq Garces, Patricia Erwin, William B Brinkman, Victor M Montori, Annie LeBlanc

**Affiliations:** 1 Knowledge and Evaluation Research (KER) Unit, Mayo Clinic, 200 First Street SW, Rochester, MN 55905, USA; 2 Mayo Medical School, Mayo Clinic College of Medicine, 200 First Street SW, Rochester, MN 55905, USA; 3 Unidad de Conocimiento y Evidencia (CONEVID), Universidad Peruana Cayetano Heredia, 430 Honorio Delgado Avenida, San Martin de Porres, Lima 31, Peru; 4 Mayo Clinic Libraries, Mayo Clinic, 200 First Street SW, Rochester, MN 55905, USA; 5 Department of Pediatrics, University of Cincinnati College of Medicine, Cincinnati Children’s Hospital Medical Center, 3333 Burnet Avenue, MLC 7035, Cincinnati, OH 45229, USA; 6 Division of Endocrinology, Diabetes, Metabolism, Nutrition, Mayo Clinic, 200 First Street SW, Rochester, MN 55905, USA; 7 Department of Health Sciences Research, Division of Health Care Policy and Research, Mayo Clinic, 200 First Street SW, Rochester, MN 55905, USA

**Keywords:** Adolescents, Children, Decision aids, Parents, Patient-centered care, Pediatrics, Shared decision making

## Abstract

**Background:**

Shared decision making in pediatrics is unique because it often involves active participation of both the child or adolescent patient and his or her caregiver(s) in the decision making process with the clinician or care team, and the extent to which the patient is involved is commensurate with their developmental level. However, little is known about the nature of pediatric-specific shared decision making interventions and their impact.

**Methods/Design:**

We will perform a systematic review with the objective of summarizing the nature of shared decision making practices, tools, techniques and technologies in the pediatric setting as well as their effects. A literature search will include Ovid MEDLINE, Ovid EMBASE, Ovid Cochrane Library, Web of Science, Scopus and Ovid PsycInfo databases in addition to consultation of a group of shared decision making experts to identify unpublished or in-progress works. We will include original research studies involving patients <18 years, their caregivers, or both, and summarize methods and approaches designed to engage participants in the health care decision making process with clinicians. Perinatal and research participation decisions will be excluded. Descriptions of participants involved, interventions used and the measured outcomes will be reported. Quality assessment will be performed according to the design of each study, where possible.

**Discussion:**

We anticipate that the paucity of published quantitative data and the heterogeneous nature of the reported results will preclude quantitative analysis. In this event, a meta-narrative approach will be undertaken.

**Trial registration:**

PROSPERO registration number: CRD42013004761

## Background

Shared decision making (SDM) is a process by which patients and clinicians engage in a partnership to synthesize the best-available medical evidence about the available options with the patient’s values, needs and preferences to arrive at the optimal decision for the patient [1]. A recent systematic review concluded that SDM tools (that is, decision aids) improve patient knowledge and patient risk perception compared to usual care [2]. Patients also reach decisions more consistent with their personal values and tend to be more involved in their decisions when a decision aid is utilized [2]. However, the extent to which SDM and decision aids can improve outcomes in pediatrics has not been summarized.

SDM in pediatrics is unique because it often involves three parties: the child or adolescent patient, the caregiver(s), and the clinician. To date, most SDM interventions have been designed with only the patient-clinician dyad in mind and do not take into account a third party. Moreover, the interaction becomes more complex in pediatrics because the role of children and adolescents as autonomous individuals capable of engaging in SDM falls on a continuum according to their developmental level [3]. Indeed, in pediatrics, the caregiver - not the patient - often is responsible for the majority of decision making.

Although pediatric decision making as a whole has been reviewed in an article by Lipstein *et al*. [4], there exists no single resource to summarize the specific topic of SDM in pediatrics with an emphasis on decision aids and other methods of patient and caregiver engagement through a systematic process. Although a systematic review of randomized controlled trials of decision aids [2] did not explicitly specify which included studies were applied in a pediatric setting, a review of titles of included studies revealed only two [5,6] that appear to be clearly applied to pediatrics (on the topics of vaccination and circumcision), although several studies covered the topic of prenatal diagnosis [7-10]. Given the paucity of randomized controlled trials of pediatric decision aids found in that systematic review, other study designs and methods of patient engagement should be explored to capture the full extent of SDM research in pediatrics.

The primary aim of this study will be to summarize the nature of SDM practices, tools, techniques and technologies in the pediatric setting as well as their effects. The secondary aim will be to describe unique differences and challenges to implementation of pediatric SDM, if there are any, in relation to SDM in adults.

## Methods/Design

### Study registration

The systematic review was registered with PROSPERO (registration number CRD42013004761; http://www.crd.york.ac.uk/PROSPERO).

### Search methods

An expert librarian will design and conduct an electronic search strategy with the input of an interdisciplinary team with expertise in SDM and conducting systematic reviews. We will search electronic databases (Ovid MEDLINE, Ovid EMBASE, Ovid Cochrane Library, Web of Science, Scopus and Ovid PsycInfo databases) from inception to current time to identify relevant studies.

We will complement our electronic search by reviewing the reference lists of the eligible primary studies. We will also conduct a manual search for systematic and narrative reviews on the topic and include articles referenced therein through the same inclusion process used for articles discovered in the primary literature search; however, the reviews themselves will not be included unless they present original, previously unpublished data. We will also consult experts, including a Facebook group of SDM experts, to identify additional works including those that are unpublished or in progress.

### Eligibility criteria

Studies targeting patients <18 years, their caregivers, or both, published from database inception to present, which describe a method or approach (including practices, tools, techniques and technologies such as SDM, decision aids and decision support tools) designed to facilitate involvement in the decision making process for medical decisions will be included. Eligible studies may be of any design, including descriptive studies, with or without comparator groups, but must be targeted to the setting of any health care decision except those decisions pertaining to antenatal care, perinatal care, or research study participation decisions. Studies will be included regardless of reported outcomes.

We will exclude studies designed to educate, motivate or change behaviors of patients without specifically engaging them in a decision making process, based on the prevailing definitions of SDM [11-13]. We will also exclude studies reporting decision making in relation to perinatal care or participation in research studies, given that their focus significantly differs from the majority of clinical decision making in pediatrics. Studies that are not English language will be excluded.

### Study selection

We will upload search results into an online reference management system (DistillerSR, Ottawa, Canada) to allow for more efficient and transparent processing with better progress tracking and real-time evaluation of inter-reviewer agreement. Two reviewers will independently consider the potential eligibility of each of the abstracts and titles from the retrieved citations and will request full text versions for these potentially eligible studies. Working separately and independently, reviewers will assess the full text of reports to confirm eligibility.

Reviewers will calibrate their judgments using a small sample of reports. When possible, disagreements will be resolved by consensus. When consensus cannot be achieved, a third-party arbiter will determine final inclusion. Agreement will be measured using the kappa or phi statistics, the latter being appropriate when the distribution of agreement is extreme.

### Data collection and extraction

Two reviewers will extract data, using a predefined data extraction form, including general information about the included studies (for example, study design, participant selection process, setting, clinical context), participants (for example, targeted participants, included ages, sociodemographics) and outcomes (all measured outcomes, including health-related outcomes, observing patient involvement (OPTION) scores [14], decisional conflict scores [15], and measures of patient knowledge and satisfaction). Given that our preliminary literature survey revealed heterogeneity in reported outcomes, we anticipate other unanticipated outcomes may be reported as well. If the extraction process reveals descriptive factors or outcomes from studies that the reviewers deem potentially useful to include and are not included in the initial data extraction form, we will collect such data preliminarily in free text form and revise the data extraction form to permit collection of the data in a coded fashion.

### Risk of bias assessment

We will conduct a quality assessment according to the design of each included study. For example, we will assess the methodological quality of randomized controlled trials using the Cochrane risk of bias tool [16] and appraise observational studies using the Newcastle-Ottawa quality assessment tool [17].

### Analysis

We will first summarize and describe the methods and approaches designed to engage participants in the decision making process with clinicians. Then we will assess the impact these SDM methods and approaches have on measures of patient engagement (such as OPTION scores), decisional conflict (such as decisional conflict scores), satisfaction, and health outcomes (such as quality of life, course of disease).

If there is enough available data, a meta-analytic approach will be taken to generate estimates of the impact of SDM interventions using Stata (StataCorp, College Station, TX, USA) to conduct the analyses.

We consider that the nature of our question, along with the lack of a standard approach to evaluate SDM across existing studies, will make the development of a quantitative meta-analysis unfeasible. If this is the case, data extracted from the included studies will be analyzed following a meta-narrative approach suggested by Greenhalgh *et al*. [18].

The meta-narrative approach involves six phases used to develop a storyline that maps the development and progression of a field. The first two phases, which have begun with the present paper, are planning and searching. The planning phase involves assembly of a multidisciplinary team with experience in the relevant fields. Our team includes four physicians (including one pediatrician), methodologists, a medical student, an epidemiologist and a librarian, all with extensive experience in the field of SDM. Together, the team outlined a broad, open-ended research question (What is the nature of SDM in pediatrics, and what unique differences and challenges are there in the implementation of pediatric SDM when compared to the adult setting?) and agreed upon the relevant outcomes (descriptions of participants and interventions, measures of SDM). The search strategy was developed with the assistance of a professional librarian (P.E.) conferring with the interdisciplinary team. References of relevant papers found in the initial literature search will be combined with informal networking to identify studies not found directly through the literature search, including those that are unpublished or in progress.

The mapping phase involves identification of key conceptual, theoretical, methodological and instrumental elements. We anticipate that three major conceptual elements will be the varying level of involvement of the patient based on his or her developmental level, involvement of a surrogate decision maker (such as the caregiver), and tryadic conversations involving the clinician, pediatric patient and caregiver. Many of the key elements, however, may be similar to those of SDM in adults (for example, role of the clinician and patient/caregiver, challenges in measurement of SDM), making the involvement of the interdisciplinary team of SDM experts crucial. Key researchers, resources and events will be mapped in a manner consistent with the prevailing terminology used in the field.

The appraisal phase involves extraction of data and grouping related data, where appropriate. The synthesis phase extracts from those studies key dimensions of pediatric SDM that have been researched and focuses on generating a narrative of how each study contributed to that dimension of pediatric SDM (for example, level of patient involvement based on developmental level, surrogate decision makers, tryadic conversations). Any apparent conflicts between studies will be highlighted and explored.

The final phase of the meta-narrative approach, ‘recommendations,’ involves a reflective dialogue with the multidisciplinary team and other stakeholders to summarize the state of the field and lessons learned. This will include comparison of SDM in pediatrics with the adult setting based on the available literature [2] and expert knowledge of members of our team. Although the meta-narrative approach suggests that practice, policy and research recommendations should be made in this section, we feel that, based on the role of the reviewers and scope of the project, explicit clinical practice and policy recommendations are not appropriate. However, suggestions for future research may be feasible based on the research background of the team.

### Publication bias

We will visually inspect asymmetry of funnel plots and conduct the Egger regression test for continuous outcomes and Peters test for binary outcomes when enough data are available and low heterogeneity exists across studies [19-22].

### Author contact

Authors of included studies will be contacted to verify data extraction and provide missing information, if available. The process for contacting authors will begin with an email to the corresponding author if an email address is available, or a phone call if only a phone number is provided. The first author will be copied if their email address is included in the manuscript. If no response is received within one week from the corresponding or first author, a follow-up email or phone call will be attempted. If a response to the follow-up email or phone call has not been received within one week, missing information will be reported as ‘not reported’.

### Reporting

If we are able to conduct a quantitative systematic review, the study will be reported according to the Preferred Reporting Items for Systematic Reviews and Meta-Analyses (PRISMA) workgroup recommendations [23]. If the meta-narrative approach is taken, the Realist and Meta-narrative Evidence Syntheses: Evolving Standards (RAMESES) publication standards will be used [24].

## Discussion

We intend for a systematic review of SDM in pediatrics to be instructive in several ways. For one, it shall be descriptive by highlighting interventions intended to facilitate SDM in the pediatric setting and serve as a guide for practicing pediatricians. Moreover, it will expose barriers and gaps in our knowledge of SDM in pediatrics to guide future research.

The greatest challenge will be the anticipated paucity of published literature and heterogeneity of reported outcomes. In an earnest attempt to address the former issue, we will directly involve known researchers in the field in addition to querying an online group of SDM experts to identify gaps in our literature search, including unpublished or in-progress studies. Given that the precise definition of SDM is a current source of debate [11-13], there is no clear consensus regarding which outcome measures are most relevant for quantifying SDM. While recognizing that quantitative approaches are preferred, we anticipate that such an analysis may not be possible, and we may need to resort to the meta-narrative approach.

We anticipate this study to be a cornerstone in pediatric SDM research, acting as a guide for practicing clinicians and a starting point for continued efforts to summarize available knowledge for pediatric SDM researchers.

## Abbreviations

OPTION: Observing patient involvement in decision making; PRISMA: Preferred Reporting Items for Systematic Reviews and Meta-Analyses; RAMESES: Realist and Meta-narrative Evidence Syntheses: Evolving Standards; SDM: Shared decision making.

## Competing interests

The authors declare that they have no competing interests.

## Authors’ contributions

KW drafted the initial protocol, developed the inclusion criteria and revised the manuscript. GP and JD contributed to the initial protocol and provided methodological support. PE assisted in development of the literature search and inclusion criteria, provided methodological support, and critically revised the manuscript. WB and AL assisted in development of the inclusion criteria, provided methodological support, and critically revised the manuscript. VM provided methodological support and critically revised the manuscript. All authors read and approved the final manuscript.
